# Wild-Type Mouse Models to Screen Antisense Oligonucleotides for Exon-Skipping Efficacy in Duchenne Muscular Dystrophy

**DOI:** 10.1371/journal.pone.0111079

**Published:** 2014-11-03

**Authors:** Limin Cao, Gang Han, Ben Gu, HaiFang Yin

**Affiliations:** Research Centre of Basic Medical Science and Department of Cell Biology, Tianjin Medical University, Heping District, Tianjin, China; Institut de Recerca de la Santa Creu i Sant Pau, Spain

## Abstract

A readily available animal model is essential for rapidly identifying effective treatments for Duchenne muscular dystrophy (DMD), a devastating neuromuscular disorder caused by the lack of dystrophin protein, which results from frame-disrupting mutations in the DMD gene. Currently, the *mdx* mouse is the most commonly used model for antisense oligonucleotide (AO)-mediated exon skipping pre-clinical studies, with a mild phenotype. However, the accessibility of *mdx* mouse colonies particularly in developing countries can constrain research. Therefore in this study we explore the feasibility of using wild-type mice as models to establish exon-skipping efficiency of various DMD AO chemistries and their conjugates. Four different strains of wild-type mice and six different AO chemistries were investigated intramuscularly and the results indicated that the same exon-skipping efficiency was achieved for all tested AOs as that from *mdx* mice. Notably, levels of exon-skipping obtained in *C57BL6* and *C3H* and *mdx* mice were most closely matched, followed by *ICR* and *BALB/C* mice. Systemic validation revealed that wild-type mice are less responsive to AO-mediated exon skipping than *mdx* mice. Our study provides evidence for the first time that wild-type mice can be appropriate models for assessing DMD AO exon-skipping efficiency with similar sensitivity to that of *mdx* mice and this finding can further accelerate the development of effective DMD AOs.

## Introduction

Duchenne muscular dystrophy (DMD) is an X-linked, lethal neuromuscular disorder caused by frame-disrupting mutations in the DMD gene, which ultimately result in the lack of functional dystrophin protein [1]. Currently there is no treatment available in clinic except for the use of steroid [2]–[4]. Numerous approaches for treating DMD are being investigated with antisense oligonucleotide (AO)-mediated exon-skipping therapeutics showing great promise from pre-clinical studies and phase II clinical trials [5]–[9]. However recent failure with Drisapersen from GSK and Prosensa phase III clinical trials highlights the importance of identifying safer and more effective AOs.

Animal models play a critical role in identifying effective treatments for DMD. Currently available DMD animal models include dystrophin-deficient mice (*mdx*) [10]–[13], dystrophin/utrophin double knock-out mice (*DKO*) [14], dystrophic dog [15], [16] and transgenic dystrophic pig [17], transgenic humanized *hDMD* mice [18]. Although recently different variants of *mdx* mice containing different mutations e.g. *mdx2cv, mdx3cv, mdx4cv, mdx5cv* and exon 52 deficient *mdx52* have emerged and been extensively used for different purposes [19]–[21]. Among them, *mdx* has been the most commonly used animal model for pre-clinical studies, particularly for DMD AO drug screen, which greatly contributes to ongoing clinical trials [10], [13], . However, the accessibility and maintenance of *mdx* mouse colonies are costly, particularly in developing countries, as *mdx* mouse colonies are not widely distributed worldwide and also require special diets and higher-standard breeding condition compared to healthy wild-type mice, which can be a burden for researchers with limited resources. In addition, although *in vitro* models substantially facilitated the optimization of AO sequences for DMD, *in vitro* models are not completely predictive of *in vivo* efficacy for some chemistry e.g. PMO (phosphorodiamidate morpholino oligomer) and PNA (peptide nucleic acid), which are notoriously difficult to transfect *in vitro*, but showing high activity *in vivo* [24]–[28]. Thus, a readily available and more cost-effective alternative *in vivo* model which allows researchers to identify uptake in different genetic background before optimization will further speed up the development of DMD AOs.

Therefore, we wished to investigate whether wild-type mice can be used to evaluate *in vivo* uptake and exon-skipping efficiency of new exon-skipping therapeutics before proceeding to optimization of a therapeutic regime in *mdx* mice or appropriate animal models. Importantly, this would lower the financial barrier of innovation in the DMD field and lead to new therapeutic molecules for DMD and other genetic diseases [29], [30]. Here we demonstrated that DMD AO exon-skipping efficacy in wild-type mice is comparable to that of *mdx* mice via local intramuscular evaluation of six different AO chemistries, though systemic validation in *C57BL6* and *C3H* with PMO revealed less effective exon skipping observed in wild-type mice than that of *mdx* mice.

## Materials and Methods

### Antisense oligonucleotide

Six different AO chemistries were evaluated in this study. PMO was purchased from GeneTools (Oregon, USA), and peptide-conjugated PMOs were synthesized with >90% purity by Sarepta Therapeutics (formerly AVI Biopharma Inc, Oregon, USA). PNA and peptide-PNA conjugates were synthesized with high-performance liquid chromatography purification to >90% purity by Panagene (Daejeon, Korea). Details of tested AOs were shown in Table 1. Different AO lengths and sequence positions with respect to the murine DMD exon 23 boundary region were shown in Table 1.

**Table 1:** Antisense oligonucleotide nomenclature.

Name	Sequence
PMO	5′- GGCCAAACCTCGGCTTACCTGAAAT - 3′
PNA	5′-GGCCAAACCTCGGCTTACCTGAAAT-3′
2′OmePS	5′-GGCCAAACCUCGGCUUACCU-3′
B-MSP-PMO	5′-RXRRBRRXRRBRB-ASSLNIAX-ggccaaacctcggcttacctgaaat-3′
MOE25(PS)	5′-GGCCAAACCTCGGCTTACCTGAAAT-3′
MSP	ASSLNIA

MSP, muscle-specific peptide and B - β-alanine [34].

### Animals and intramuscular injection of AOs

Four different strains of wild-type mice including *C57BL6*, *C3H*, *BALB/C* and *ICR*, and *mdx* mice were used in all experiments (3 mice each group). The experiments were carried out in the animal unit, Tianjin Medical University (Tianjin, China) according to procedures authorized and specifically approved by the institutional ethical committee (Permit Number: SYXK 2009–0001). For intramuscular studies, TA (tibialis anterior) muscles of 6-8-week old experimental mouse were injected with various amounts of AOs in 40 µl saline solution. For systemic injections, PMOs dissolved in 100 µl saline buffer were injected into tail vein of *C57BL6*, *C3H* and *mdx* mice at the final dose of 25 mg/kg. In order to examine the duration of dystrophin expression induced by various AOs, mice were killed by cervical dislocation at desired time-points i.e. 48 hr, 2-week and 4-week after injection, and muscles tissues were snap-frozen in dry ice-cooled isopentane and stored at −80°C.

### RNA extraction and nested RT-PCR analysis

Total RNA was extracted from tested muscle tissues with Trizol reagent as per manufacturer's protocol (Invitrogen, UK) and 400 ng of RNA template was used for 20 µl RT–PCR with OneStep RT– PCR kit (Qiagen, UK). The primer sequences for the initial RT-PCR were Exon20Fo: 5′-CAGAATTCTGCCAATTGCTGAG-3′and Exon26Ro: 5′-TTCTTCAGCTTGTGTCATCC-3′for amplification of mRNA from exons 20 to 26. The cycle conditions were 95°C for 30 seconds, 55°C for 30 seconds, and 72°C for 2 minutes for 25 cycles. The RT-PCR product (0.8 µl) was then used for a nested PCR performed in 20 µl with 0.5 U Taq DNA polymerase (Invitrogen, UK). The primer sequences for the second round were Exon20F1: 5′-CCCAGTCTACCACCCTATCAGAGC-3′ and Exon 24R1: 5 ′-CCTGCCTTTAAGGCTTCCTT-3 ′. The cycle conditions were 94°C for 30 seconds, 57°C for 30 seconds, and 72°C for 2 minutes for 25 cycles. The products were examined by electrophoresis on a 2% agarose gel. The quantification of full-length and skipped RT-PCR bands was based on densitometry of gel images and analyzed using ImageJ software (http://rsbweb.nih.gov/ij/).

### cDNA synthesis

cDNA synthesis was carried out using Applied Biosystems High capacity cDNA reverse transcription kit. Reactions contained 2 µg RNA, 1× RT Buffer, 4 mM dNTP mix, RT random primers and MultiScribe reverse transcriptase in a final volume of 20 µl. The reaction was performed in a Techne TC-412 thermo cycler: 25°C for 10 min, 37°C for 120 min, and 85°C for 5 min.

### Quantitative PCR

Real-time PCR were performed using an Applied Biosystems StepOnePlus qPCR System with SYBR Green dye. Reactions were carried out in 20 µl reactions containing 100 ng cDNA template, 2× master mix (2× reaction buffer, 0.025 U/µl Taq polymerase, 5 mM MgCl2 and 200 µM dNTP) and 250 nM primers. Cycling was initiated at 95°C for 10 min, followed by 40 cycles of 95°C for 15 s, 60°C for 1 min. Fluorescence was measured at the end of the elongation phase in each cycle. The homogeneity of amplicons was verified by melt curve.

Primers used to measure total DMD transcripts were designed within exons 20 and 21: (exon 20 forward: 5′ ACC-ACC-CTA-TCA-GAG-CCA-AC and exon 21 reverse: 5′CAG-AAA-CAT-TGG-CCC-CTG-TC). Exon23-containing transcripts were amplified by exon 23 forward (5′ ATT-GAG-GGG-CAC-TGG-AAG-AA) and reverse primer spanning exon 24 (5′ ACA-TCA-ACT-TCA-GCC-ATC-CA).

### Hematoxylin and eosin staining

Routine haematoxylin and eosin staining was used to examine overall muscle morphology.

### Statistical analysis

All data are reported as the mean ±SEM. Statistical differences between different treated groups were evaluated by SigmaStat (Systat Software Inc. Chicago, IL, USA) and Mann-Whitney Rank Sum Test and Kruskal-Wallis One Way Analysis of Variance on Ranks were applied.

## Results

### *C57BL6* mice are good models for assessing DMD AO exon skipping efficacy

**Figure 1:**
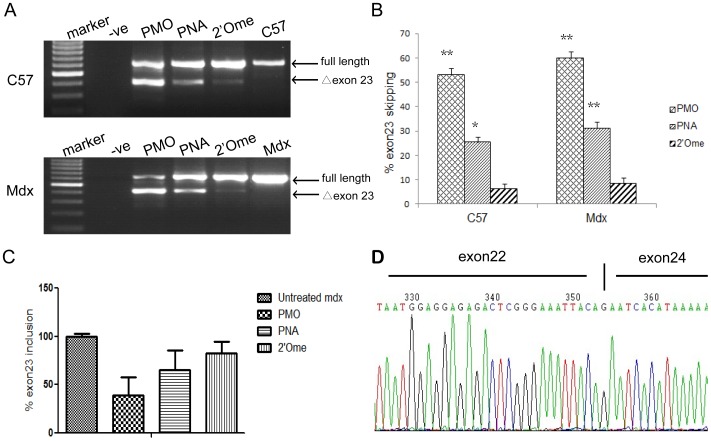
Effective exon skipping in *C57BL6* and *mdx* mice by local intramuscular injection of different AOs. (A) RT-PCR for detecting exon skipping at the RNA level with treated TA muscles 48 hr after intramuscular injection of 2 µg PMO, 5 µg PNA and 5 µg 2′Ome PS in *C57BL6* and *mdx* mice. The numbered Δexon23 is for exon 23 skipping. –ve stands for RT-PCR blank control and the same below unless otherwise specified. (B) Quantitative analysis of exon skipping induced by different AOs in *C57BL6* and *mdx* mice (**P<0.001; *P<0.05, n = 3). (C) Quantitative PCR validation for detecting exon skipping at the RNA level with treated TA muscles 48 hr after intramuscular injection in *mdx* mice. (D) Sequence analysis of the exon 23 skipped band in the RT-PCR products.

To evaluate the feasibility of *C57BL6* as an *in vivo* test system for AO-mediated exon skipping, we chose three well-studied and representative AO chemistries including PMO (phosphodiamidate morpholino oligomer), PNA (peptide nucleic acid) and 2′Ome PS (2′O-methyl RNA with phosphorothioate backbone) in our study [25], [26], [28], [31]–[33]. Two microgram of PMO, 5 µg of PNA or 5 µg of 2′Ome PS was injected into TA muscles of adult *C57BL6* mice and exon 23 skipping at RNA level was evaluated 48 hr after injection. RT-PCR results revealed that effective exon skipping can be achieved in *C57BL6* with PMO and PNA, approximately 53.1% and 25.4%, respectively (Fig.1A and B). Compared to PMO and PNA, much less efficient exon skipping was detected with 2′Ome PS, with only about 7.7% (Fig. 1B). Subsequent sequencing confirmed exon 23 exclusion in murine DMD gene (Fig. 1D). When compared with *mdx* mice (Fig. 1B), comparable level of exon skipping was achieved in *C57BL6* for all tested AOs with the same order of efficiency, in which PMO showed the highest activity, followed by PNA and 2′Ome PS. Subsequent quantitative PCR results corroborated with the RT-PCR data and demonstrated the same pattern of exon skipping efficiency for PMO, PNA and 2′Ome PS in *mdx* mice (Fig. 1C). Consistently, further evaluations of PMO and PNA derivatives e.g. B-MSP-PMO (B refers to B peptide and MSP represents muscle-specific peptide), PNA-MSP and MOE PS (2′O-Methoxyethyl with phosphorothioate backbone), which were evaluated in *mdx* mice previously [25], [27], [34], [35], in *C57BL6* revealed that effective exon skipping can be induced in *C57BL6* at 48 hr after local intramuscular injection, with B-MSP-PMO being better than PNA-MSP and MOE PS (Figs. S1A and B), suggesting that *C57BL6* is a viable animal model for DMD AO screen.

### Similar duration of exon skipping effects can be established between *C57BL6* and *mdx* mice

**Figure 2:**
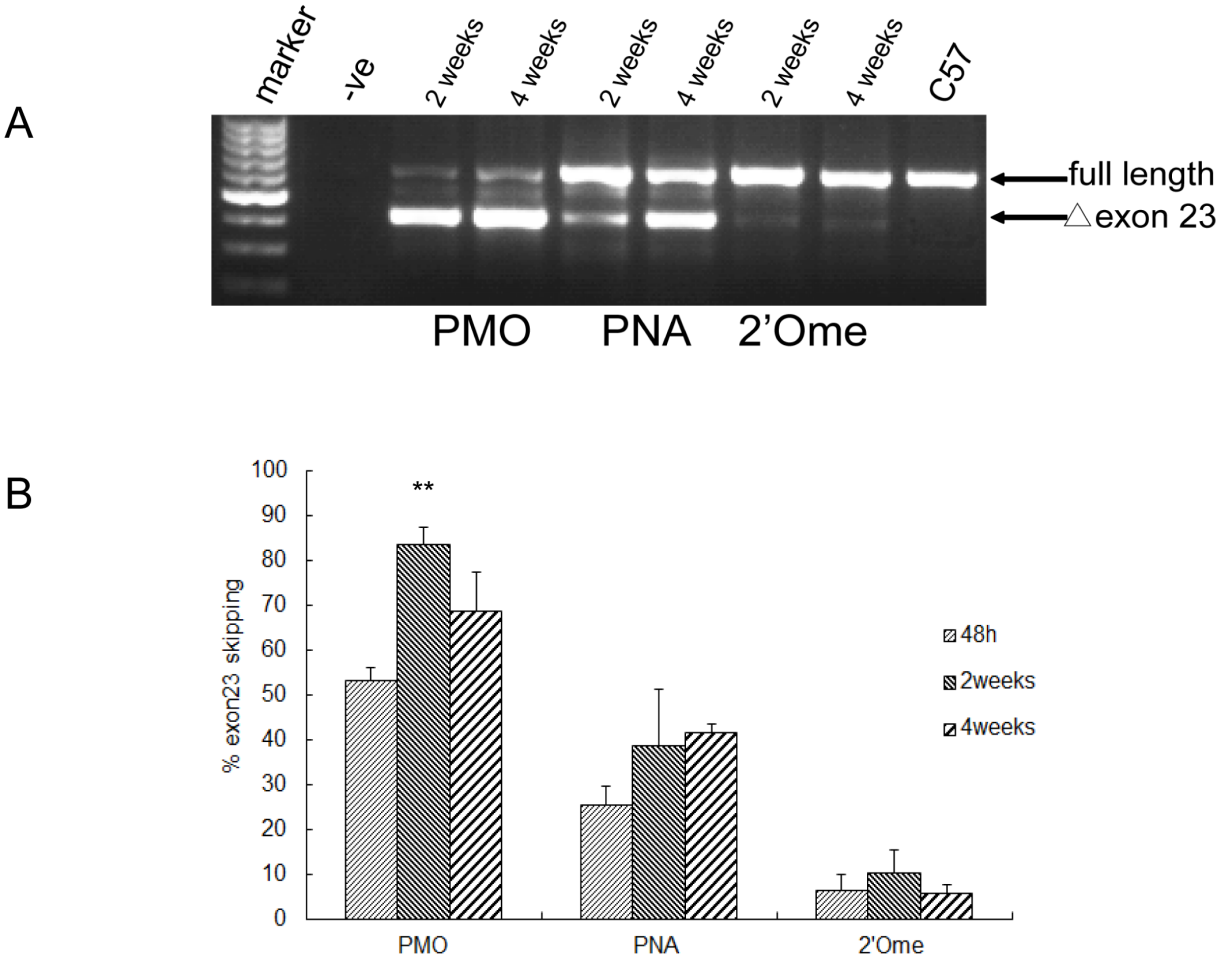
Duration of exon skipping effects in *C57BL6* with different AOs by local intramuscular injection. (A) RT-PCR results for PMO, PNA and 2′Ome PS in *C57BL6* at different time-points e.g. 2 and 4 weeks after local intramuscular injection. (B) Quantification of exon 23 skipping efficiency for PMO, PNA and 2′Ome PS at different time-points (**p<0.001, n = 3).

Given effective exon skipping observed in *C57BL6* with PMO, PNA and 2′Ome PS, we wished to examine whether the similar duration of exon skipping effects can be induced in *C57BL6* to that of *mdx* mice as reported earlier [26], [27]. The same amount of PMO (2 µg) and PNA, 2′Ome PS (5 µg) was injected into TA muscles of adult *C57BL6* mice, respectively, and tissues were harvested at different time-points i.e. 2- and 4-week post-injection. Subsequent RT-PCR revealed almost complete exon 23 exclusion was obtained in TA muscles treated with PMO at 2-week which decreased at 4-week time-point, suggesting the peak time for measuring PMO exon skipping is 2-week post-injection. For PNA, consistent with our previous observation in *mdx* mice [25], PNA was higher at the 4-week time-point in *C57BL6*, with about 41.3% exon 23 skipping (Fig. 2A and B). In contrast, there is no significant difference in exon skipping efficiency detected for 2′Ome PS between 2- and 4-week time-points in *C57BL6*, with 2-week time-point (9.5%) showing a marginal increase compared to 4-week time-point (5.8%), corroborating with the earlier report [26]. Consistent with the data from 48 hr time-point, 2′Ome PS shows much less activity than those of PMO and PNA. In addition, no inflammation was observed in treated TA muscles at any time-point tested as examined by Hematoxylin & Eosin staining (Fig. S2). Overall, similar duration of exon skipping effects was established in *C57BL6* to that of *mdx* mice, further supporting *C57BL6* is a good model system for DMD AO screen.

### Other wild-type strains are viable model systems for screening DMD AO

**Figure 3:**
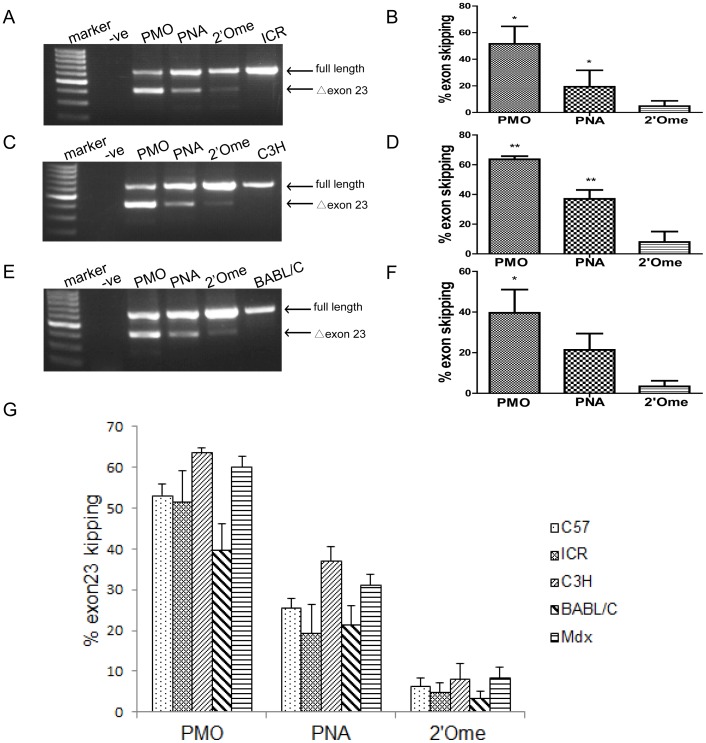
Efficient exon skipping in other wild-type mice by local intramuscular injection. (A) RT-PCR results for PMO, PNA and2′Ome PS in *C3H* mice 48 hr after local intramuscular injection. The numbered Δexon23 is for exon 23 skipping. (B) Quantitative analysis of exon skipping induced by different AOs in *C3H* mice (*P<0.05, n = 3). (C) RT-PCR results for PMO, PNA and 2′Ome PS in *ICR* mice 48 hr post-injection. (D) Quantitative analysis of exon skipping efficiency by different AOs in *ICR* mice (**P<0.001, n = 3). (E) RT-PCR results for PMO, PNA and 2′Ome PS in *BABL/C* mice 48 hr post-injection. (F) Quantitative analysis of exon skipping induced by different AOs in *BABL/C* mice (*P<0.05, n = 3). (G) Cross-comparison between different wild-type and *mdx* mice per AO chemistry.

Since effective exon skipping was achieved in *C57BL6* with comparable level and similar duration of effects to that of *mdx* mice, we extended our studies to other common laboratory strains including *ICR, C3H* and *BALB/C*. To examine the possibility and applicability of these mice as alternatives to *mdx* mice for DMD AO screen, the same AOs including PMO, PNA and 2′Ome PS were injected into TA muscles of these three different strains of mice, respectively, under the same dosing regimen as did with *C57BL6*. Treated TA muscles were harvested 48 hr after injection and assayed with RT-PCR. RT-PCR results indicated that significant exon skipping was induced for these three wild-type mice with all tested AOs (Fig. 3). Notably, the same order of exon skipping efficiency was achieved in these wild-type mice as those from *C57BL6* and *mdx* mice, with PMO showing the highest activity, followed by PNA and 2′Ome PS in *ICR*, *C3H* and *BALB/C* mice, respectively (Fig. 3B, D and F), suggesting these wild-type mice are applicable for DMD AO screen and there is no major differences in uptake and efficacy of the AOs in between the strains.

Furthermore we cross-compared the exon skipping efficiency between four wild-type and *mdx* mice based on the same AO chemistry. In regards to PMO, which demonstrated the highest level of exon skipping compared to PNA and 2′Ome PS in any tested animal model, similar exon skipping efficiency was achieved between *C3H* and *mdx* mice with the mean value of 63.7% and 60%, respectively (Fig. 3). Second to *C3H*, significant exon skipping was detected in *C57BL6* and *ICR*, approximately 53.1% and 58%, respectively (Fig. 3G). Although slightly lower level of exon skipping was detected in *BALB/C* mice, the difference was not significant compared to *mdx* mice. Of note, similar pattern was observed for PNA and 2′Ome PS, with comparable exon skipping achieved between *C3H* and *mdx* mice, followed by *C57BL6* and *ICR* and the least exon skipping detected in *BALB/C* mice, though the overall level for these two chemistries is lower than that of PMO.

Intravenous delivery of PMO in *C57BL6* and *C3H*, at a low dose of 25 mg/kg for 3 weekly injections, revealed that detectable level of exon skipping was observed in quadriceps, abdominal and TA from treated *C57BL6* and *C3H*, though the overall level is lower than counterparts from treated *mdx* mice under identical dosing regimen (Fig. S3). These data indicate that wild-type mice are responsive to AO-mediated exon skipping systemically, though to a less extent than that of *mdx* mice.

Taken together, our study demonstrated that wild-type mice can be used as alternative animal models for DMD AO screen and a means to ascertain variation in efficacy of AOs in mice with different genetic backgrounds, which can inform about the potential variation in therapeutic efficacy that might be present in the patient population.

## Discussion

AO-mediated exon skipping therapeutics offers hope for DMD patients based on the outcome from local and systemic trials in human subjects [7]–[9], [23], [36], [37]. However, to further accelerate the development of effective treatments for DMD, a reliable and cost-effective animal model is a critical component among others. In this report, we tested the feasibility and applicability of wild-type mice, which are easily accessible and require low maintenance cost, as *in vivo* models to evaluate DMD AO efficacy and distribution prior to studies in relevant disease models. This study implies that *in vivo* efficacy of AOs, which may not correlate to efficacy in cell culture, can also be used in wild-type mice for other exons in DMD in which specific models do not exist and even for other genes and for AOs of other functions.

Compared to the *in vitro* system, *in vivo* model presents with properly-assembled musculature including the presence of connective tissue and fibroblasts et al. More pertinently, transfection reagents are frequently required to deliver the AOs in cell culture while these could substantially mask the authentic property of tested AOs. Thus, *in vitro* efficacy does not reflect *in vivo* efficacy. This notion is supported by previous studies, in which 2′Ome PS showed the greatest exon skipping activity *in vitro* compared to PMO and PNA but the least effective one *in vivo* locally and systemically [24], [27], [38]. In the current study, we reaffirmed findings from previous studies in *mdx* mice that demonstrated better *in vivo* exon skipping efficacy of PMO and PNA compared to negatively charged RNA analog -2′Ome PS, which can be attributed to their higher serum stability and sequence-specificity [27], [31]. Peptide modifications, such as cell-penetrating peptides (CPPs) and targeting motifs are also more accurately assessed *in vivo*, irrespective of wild-type or *mdx* mice [34], [39], [40]. Our findings further emphasize the importance of *in vivo* screen for DMD AOs, particularly for neutral PMO and PNA, which are notoriously difficult to transfect *in vitro*. Moreover, *in vivo* screen in wild-type mice can be used as a rapid drug screening platform as 48 hr post-injection is sufficient to differentiate the potency of different AOs.

Although systemic evaluation of PMO in wild-type mice did not turn out as efficient as that from *mdx* mice under identical dosing regimens, detectable level of exon skipping was observed in *C57BL6* and *C3H* with a low dose of PMO, suggesting wild-type mice, particularly *C57BL6* and *C3H*, are viable animal models for systemic evaluation of DMD AOs. We speculate the difference between wild-type strains and *mdx* mice can be attributed to the condition of muscle membrane e.g. membrane leakage in *mdx* mice, which likely impacts on systemic delivery more than local administration by facilitating the vascular escape of AOs. In addition, we chose intravenous administration for our systemic studies since that it was reported previously that intravenous injection led to higher level of AO detention in body-wide tissues and increased exon skipping efficiency in muscles compared to intraperitoneal and subcutaneous administration in *mdx* mice [41]. Furthermore, in our earlier report, we carried out a side-by-side comparison between intravenous and intraperitoneal injections in dystrophin/utrophin double knock-out mice (*DKO*) and the data demonstrated that intravenous administration is more effective in enhancing AO-mediated exon skipping than intraperitoneal injection [42]. Therefore, the overall low level of exon skipping observed in systemic studies is likely due to the low dose applied and unlikely related to the delivery routes used.

Ultimately, except for the *hDMD* mouse [18], all mouse models are primarily used to evaluate effects of backbone chemistries, peptide modifications and dosing regimens on delivery efficiency rather than efficacy of specific sequences because of sequence differences between human and murine *DMD* genes. Functional improvement in DMD mice, while useful in previous studies to demonstrate that restoration of dystrophin expression at a specific level can lead to functional improvement, is not as crucial for the above-mentioned uses for a variety of reasons. First, these mice do not fully recapitulate human pathology, so functional improvements may not be translatable; second, the dystrophin restored by exon skipping in humans and mice have different domains removed so it may have different functional effects; third, sequence-specific differences may have a bigger role to play on exon skipping once the AO is within the cell, thus the inability to test human sequences in mice means the results are not directly translatable. Moreover, the differences in systemic distribution between wild-type strains may provide insights into the variation that will be present in human patients and can inform better designs for AOs. Thus, our study indicates that it is beneficial financially and therapeutically to evaluate AO chemistry and modifications in wild-type strains despite the inability to assess functional improvements.

## Conclusions

In summary, we demonstrated that wild-type mice are a practicable system for DMD AO screen locally and systemically, with wild-type mice showing less responsive to AO-mediated exon skipping than *mdx* mice systemically. The application of this model system can potentially open up a new avenue for DMD study and further accelerate the development of DMD AOs for the treatment of DMD patients.

## Supporting Information

Figure S1 Evaluation of other AO chemistries in *C57BL6* mice intramuscularly. (**A**) RT-PCR for detecting exon skipping at the RNA level with treated TA muscles 48 hr after intramuscular injection of 2 µg B-MSP-PMO, 5 µg PNA-MSP and 5 µg MOE. The numbered Δexon23 is for exon 23 skipping. (B) Quantitative evaluation of exon skipping induced by different AOs in *C57BL6* (**P<0.001 and *P<0.05, n = 3).
(TIF)

Figure S2 Routine hematoxylin and eosin staining for examining muscle morphology. Hematoxylin and eosin staining of TA tissue sections from treated *C57BL6* mice with 2 µg PMO, 5 µg PNA and 5 µg 2′Ome PS by local injection at different time-points e.g. 48 hr, 2 and 4 weeks after injection, and *C57BL6* normal controls. Scale Bar  = 100 µm. No difference was observed between treated and untreated *mdx* mice.
(TIF)

Figure S3 Systemic evaluation of PMO in wild-type and *mdx* mice. (A) RT-PCR results for systemic validation in *C57BL6*, *C3H* and *mdx* mice with PMO intravenously, at a low dose of 25mg/kg for 3 weekly injections. The numbered Δexon23 is for exon 23 skipping. (B) Quantitative evaluation of exon skipping in body-wide muscles in treated *C57BL6, C3H* and *mdx* mice (**P<0.001, n = 3).
(TIF)

## References

[1] Hoffman EP, Knudson CM, Campbell KP, Kunkel LM (1987) Subcellular fractionation of dystrophin to the triads of skeletal muscle. Nature 330: 754–758. 2447503 10.1038/330754a0

[2] Angelini C, Peterle E (2012) Old and new therapeutic developments in steroid treatment in Duchenne muscular dystrophy. Acta Myol 31: 9–15. 22655511 PMC3440806

[3] Bhattacharya S, Nayak A, Schiavone M, K Bhattacharya S (2004) Solubilization and concentration of carbon dioxide: novel spray reactors with immobilized carbonic anhydrase. Biotechnol Bioeng 86: 37–46. 15007839 10.1002/bit.20042

[4] Tidball JG, Wehling-Henricks M (2004) Evolving therapeutic strategies for Duchenne muscular dystrophy: targeting downstream events. Pediatr Res 56: 831–841. 15531741 10.1203/01.PDR.0000145578.01985.D0

[5] Cirak S, Arechavala-Gomeza V, Guglieri M, Feng L, Torelli S, et al (2011) Exon skipping and dystrophin restoration in patients with Duchenne muscular dystrophy after systemic phosphorodiamidate morpholino oligomer treatment: an open-label, phase 2, dose-escalation study. Lancet 378: 595–605. 21784508 10.1016/S0140-6736(11)60756-3 PMC3156980

[6] Goemans NM, Tulinius M, van den Akker JT, Burm BE, Ekhart PF, et al (2011) Systemic administration of PRO051 in Duchenne's muscular dystrophy. N Engl J Med 364: 1513–1522. 21428760 10.1056/NEJMoa1011367

[7] Kinali M, Arechavala-Gomeza V, Feng L, Cirak S, Hunt D, et al (2009) Local restoration of dystrophin expression with the morpholino oligomer AVI-4658 in Duchenne muscular dystrophy: a single-blind, placebo-controlled, dose-escalation, proof-of-concept study. Lancet Neurol 8: 918–928. 19713152 10.1016/S1474-4422(09)70211-X PMC2755039

[8] Goyenvalle A, Seto JT, Davies KE, Chamberlain J (2011) Therapeutic approaches to muscular dystrophy. Hum Mol Genet 20: R69–78. 21436158 10.1093/hmg/ddr105 PMC3095062

[9] Extance A (2009) Targeting RNA: an emerging hope for treating muscular dystrophy. Nat Rev Drug Discov 8: 917–918. 19949390 10.1038/nrd3069

[10] Ahmad A, Brinson M, Hodges BL, Chamberlain JS, Amalfitano A (2000) Mdx mice inducibly expressing dystrophin provide insights into the potential of gene therapy for duchenne muscular dystrophy. Hum Mol Genet 9: 2507–2515. 11030755 10.1093/hmg/9.17.2507

[11] Allamand V, Campbell KP (2000) Animal models for muscular dystrophy: valuable tools for the development of therapies. Hum Mol Genet 9: 2459–2467. 11005802 10.1093/hmg/9.16.2459

[12] Mann CJ, Honeyman K, Cheng AJ, Ly T, Lloyd F, et al (2001) Antisense-induced exon skipping and synthesis of dystrophin in the mdx mouse. Proc Natl Acad Sci U S A 98: 42–47. 11120883 10.1073/pnas.011408598 PMC14541

[13] Wu B, Lu P, Cloer C, Shaban M, Grewal S, et al (2012) Long-term rescue of dystrophin expression and improvement in muscle pathology and function in dystrophic mdx mice by peptide-conjugated morpholino. Am J Pathol 181: 392–400. 22683468 10.1016/j.ajpath.2012.04.006 PMC3409432

[14] Rafael JA, Townsend ER, Squire SE, Potter AC, Chamberlain JS, et al (2000) Dystrophin and utrophin influence fiber type composition and post-synaptic membrane structure. Hum Mol Genet 9: 1357–1367. 10814717 10.1093/hmg/9.9.1357

[15] Sharp NJ, Kornegay JN, Van Camp SD, Herbstreith MH, Secore SL, et al (1992) An error in dystrophin mRNA processing in golden retriever muscular dystrophy, an animal homologue of Duchenne muscular dystrophy. Genomics 13: 115–121. 1577476 10.1016/0888-7543(92)90210-j

[16] Howell JM, Fletcher S, Kakulas BA, O'Hara M, Lochmuller H, et al (1997) Use of the dog model for Duchenne muscular dystrophy in gene therapy trials. Neuromuscul Disord 7: 325–328. 9267846 10.1016/s0960-8966(97)00057-6

[17] Klymiuk N, Blutke A, Graf A, Krause S, Burkhardt K, et al (2013) Dystrophin-deficient pigs provide new insights into the hierarchy of physiological derangenments of dystrophic muscle. Hum Mol Genet 22(21): 4368–82. 23784375 10.1093/hmg/ddt287

[18] Bremmer-Bout M, Aartsma-Rus A, de Meijer EJ, Kaman WE, Janson AA, et al (2004) Targeted exon skipping in transgenic hDMD mice: A model for direct preclinical screening of human-specific antisense oligonucleotides. Mol Ther 10: 232–240. 15294170 10.1016/j.ymthe.2004.05.031

[19] Araki E, Nakamura K, Nakao K, Kameya S, Kobayashi O, et al (1997) Targeted disruption of exon 52 in the mouse dystrophin gene induced muscle degeneration similar to that observed in Duchenne muscular dystrophy. Biochem Biophys Res Commun 238: 492–497. 9299538 10.1006/bbrc.1997.7328

[20] Im WB, Phelps SF, Copen EH, Adams EG, Slightom JL, et al (1996) Differential expression of dystrophin isoforms in strains of mdx mice with different mutations. Hum Mol Genet 5: 1149–1153. 8842734 10.1093/hmg/5.8.1149

[21] Aoki Y, Nakamura A, Yokota T, Saito T, Okazawa H, et al (2010) In-frame dystrophin following exon 51-skipping improves muscle pathology and function in the exon 52-deficient mdx mouse. Mol Ther 18: 1995–2005. 20823833 10.1038/mt.2010.186 PMC2990521

[22] Grounds MD, Radley HG, Lynch GS, Nagaraju K, De Luca A (2008) Towards developing standard operating procedures for pre-clinical testing in the mdx mouse model of Duchenne muscular dystrophy. Neurobiol Dis 31: 1–19. 18499465 10.1016/j.nbd.2008.03.008 PMC2518169

[23] van Deutekom JC, Janson AA, Ginjaar IB, Frankhuizen WS, Aartsma-Rus A, et al (2007) Local dystrophin restoration with antisense oligonucleotide PRO051. N Engl J Med 357: 2677–2686. 18160687 10.1056/NEJMoa073108

[24] Wang Q, Yin H, Camelliti P, Betts C, Moulton H, et al (2010) In vitro evaluation of novel antisense oligonucleotides is predictive of in vivo exon skipping activity for Duchenne muscular dystrophy. J Gene Med 12: 354–364. 20235089 10.1002/jgm.1446

[25] Alter J, Lou F, Rabinowitz A, Yin H, Rosenfeld J, et al (2006) Systemic delivery of morpholino oligonucleotide restores dystrophin expression bodywide and improves dystrophic pathology. Nat Med 12: 175–177. 16444267 10.1038/nm1345

[26] Lu QL, Mann CJ, Lou F, Bou-Gharios G, Morris GE, et al (2003) Functional amounts of dystrophin produced by skipping the mutated exon in the mdx dystrophic mouse. Nat Med 9: 1009–1014. 12847521 10.1038/nm897

[27] Yin H, Betts C, Saleh AF, Ivanova GD, Lee H, et al (2010) Optimization of peptide nucleic acid antisense oligonucleotides for local and systemic dystrophin splice correction in the mdx mouse. Mol Ther 18: 819–827. 20068555 10.1038/mt.2009.310 PMC2848838

[28] Yin H, Lu Q, Wood M (2008) Effective exon skipping and restoration of dystrophin expression by peptide nucleic acid antisense oligonucleotides in mdx mice. Mol Ther 16: 38–45. 17968354 10.1038/sj.mt.6300329

[29] Islamian JP, Mohammadi M, Baradaran B (2014) Inhibition of human esophageal squamous cell carcinomas by targeted silencing of tumor enhancer genes: an overview. Cancer Biol Med 11: 78–85. 25009749 10.7497/j.issn.2095-3941.2014.02.002 PMC4069799

[30] Zheng H, Liu JY, Song FJ, Chen KX (2013) Advances in circulating microRNAs as diagnostic and prognostic markers for ovarian cancer. Cancer Biol Med 10: 123–130. 24379986 10.7497/j.issn.2095-3941.2013.03.001 PMC3860338

[31] Heemskerk HA, de Winter CL, de Kimpe SJ, van Kuik-Romeijn P, Heuvelmans N, et al (2009) In vivo comparison of 2′-O-methyl phosphorothioate and morpholino antisense oligonucleotides for Duchenne muscular dystrophy exon skipping. J Gene Med 11: 257–266. 19140108 10.1002/jgm.1288

[32] Kendall GC, Mokhonova EI, Moran M, Sejbuk NE, Wang DW, et al (2012) Dantrolene enhances antisense-mediated exon skipping in human and mouse models of Duchenne muscular dystrophy. Sci Transl Med 4: 164ra160. 10.1126/scitranslmed.3005054 23241744

[33] Geary RS, Watanabe TA, Truong L, Freier S, Lesnik EA, et al (2001) Pharmacokinetic properties of 2′-O-(2-methoxyethyl)-modified oligonucleotide analogs in rats. J Pharmacol Exp Ther 296: 890–897. 11181921

[34] Yin H, Moulton HM, Betts C, Seow Y, Boutilier J, et al (2009) A fusion peptide directs enhanced systemic dystrophin exon skipping and functional restoration in dystrophin-deficient mdx mice. Hum Mol Genet 18: 4405–4414. 19692354 10.1093/hmg/ddp395

[35] Yang L, Niu H, Gao X, Wang Q, Han G, et al (2013) Effective exon skipping and dystrophin restoration by 2′-o-methoxyethyl antisense oligonucleotide in dystrophin-deficient mice. PLoS One 8: e61584. 23658612 10.1371/journal.pone.0061584 PMC3637291

[36] Kang JK, Malerba A, Popplewell L, Foster K, Dickson G (2011) Antisense-induced myostatin exon skipping leads to muscle hypertrophy in mice following octa-guanidine morpholino oligomer treatment. Mol Ther 19: 159–164. 20924365 10.1038/mt.2010.212 PMC3017443

[37] Goyenvalle A, Davies KE (2011) Challenges to oligonucleotides-based therapeutics for Duchenne muscular dystrophy. Skelet Muscle 1: 8. 21798085 10.1186/2044-5040-1-8 PMC3156649

[38] Malerba A, Sharp PS, Graham IR, Arechavala-Gomeza V, Foster K, et al (2011) Chronic systemic therapy with low-dose morpholino oligomers ameliorates the pathology and normalizes locomotor behavior in mdx mice. Mol Ther 19: 345–354. 21102560 10.1038/mt.2010.261 PMC3034854

[39] Yin H, Saleh AF, Betts C, Camelliti P, Seow Y, et al (2011) Pip5 transduction peptides direct high efficiency oligonucleotide-mediated dystrophin exon skipping in heart and phenotypic correction in mdx mice. Mol Ther 19: 1295–1303. 21505427 10.1038/mt.2011.79 PMC3128823

[40] Moulton HM, Moulton JD (2010) Morpholinos and their peptide conjugates: therapeutic promise and challenge for Duchenne muscular dystrophy. Biochim Biophys Acta 1798: 2296–2303. 20170628 10.1016/j.bbamem.2010.02.012

[41] Heemskerk H, de Winter C, van Kuik P, Heuvelmans N, Sabatelli P, et al (2010) Preclinical PK and PD studies on 2′-O-methyl-phosphorothioate RNA antisense oligonucleotides in the mdx mouse model. Mol Ther 18: 1210–1217. 20407428 10.1038/mt.2010.72 PMC2889733

[42] Crisp A, Yin H, Goyenvalle A, Betts C, Moulton HM, et al (2011) Diaphragm rescue alone prevents heart dysfunction in dystrophic mice. Hum Mol Genet 20: 413–421. 21062902 10.1093/hmg/ddq477

