# Correction: Wild-Type Mouse Models to Screen Antisense Oligonucleotides for Exon-Skipping Efficacy in Duchenne Muscular Dystrophy

**DOI:** 10.1371/journal.pone.0207817

**Published:** 2018-11-15

**Authors:** Limin Cao, Gang Han, Ben Gu, HaiFang Yin

S2 Fig contains incorrectly duplicated panels. The authors have provided a corrected version here.

## Supporting information

S2 Fig Routine hematoxylin and eosin staining for examining muscle morphology. Hematoxylin and eosin staining of TA tissue sections from treated *C57BL6* mice with 2 μg PMO, 5 μg PNA and 5 μg 2′Ome PS by local injection at different time-points e.g. 48 hr, 2 and 4 weeks after injection, and *C57BL6* normal controls. Scale Bar = 100 μm. No difference was observed between treated and untreated *mdx* mice.
(TIF)
