# Correction: Correction: Wild-Type Mouse Models to Screen Antisense Oligonucleotides for Exon-Skipping Efficacy in Duchenne Muscular Dystrophy

**DOI:** 10.1371/journal.pone.0212820

**Published:** 2019-02-20

**Authors:** The PLOS ONE Staff

There is an error in the Correction published on November 15, 2018. In the last sentence of the caption for S2 Fig, “*mdx*” should be “*C57BL6*.” The publisher apologizes for the error. The correct sentence is: No difference was observed between treated and untreated *C57BL6* mice.

Please view S2 Fig and the complete, correct S2 Fig caption below.

## Supporting information

S2 Fig Routine hematoxylin and eosin staining for examining muscle morphology. Hematoxylin and eosin staining of TA tissue sections from treated *C57BL6* mice with 2 µg PMO, 5 µg PNA and 5 µg 2′Ome PS by local injection at different time-points e.g. 48 hr, 2 and 4 weeks after injection, and *C57BL6* normal controls. Scale Bar   =  100 µm. No difference was observed between treated and untreated *C57BL6* mice.
(TIF)
