# Supplementary figures and images for: Wild-Type Mouse Models to Screen Antisense Oligonucleotides for Exon-Skipping Efficacy in Duchenne Muscular Dystrophy
Source: PLoS One. 2014 Nov 3;9(11):e111079. doi: 10.1371/journal.pone.0111079 (PMC4217760; doi:10.1371/journal.pone.0111079)

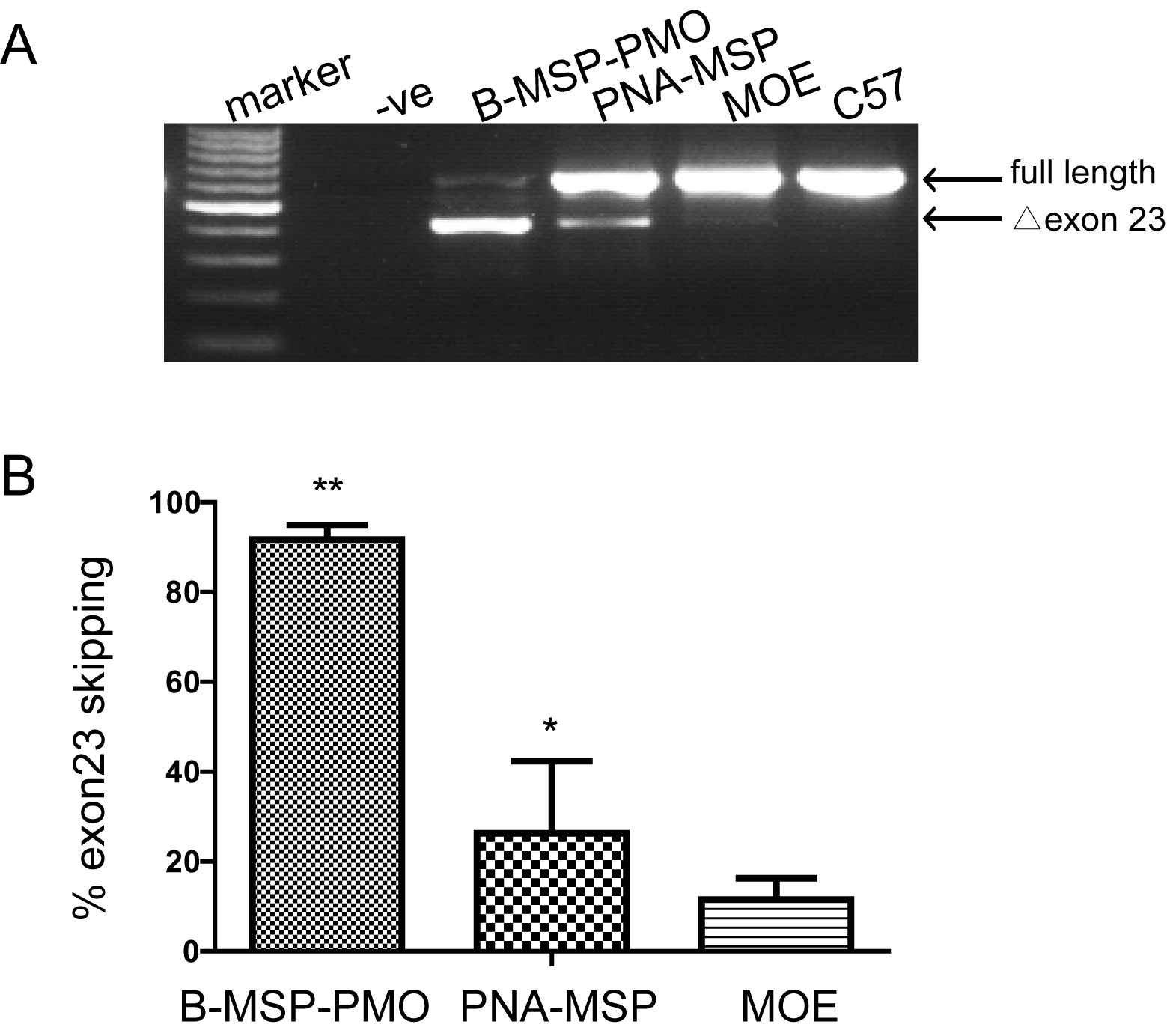

Supplement: Figure S1 — Evaluation of other AO chemistries in C57BL6 mice intramuscularly. (A) RT-PCR for detecting exon skipping at the RNA level with treated TA muscles 48 hr after intramuscular injection of 2 µg B-MSP-PMO, 5 µg PNA-MSP and 5 µg MOE. The numbered Δexon23 is for exon 23 skipping. (B) Quantitative evaluation of exon skipping induced by different AOs in C57BL6 (**P<0.001 and *P<0.05, n = 3). (TIF) [file pone.0111079.s001.tif]

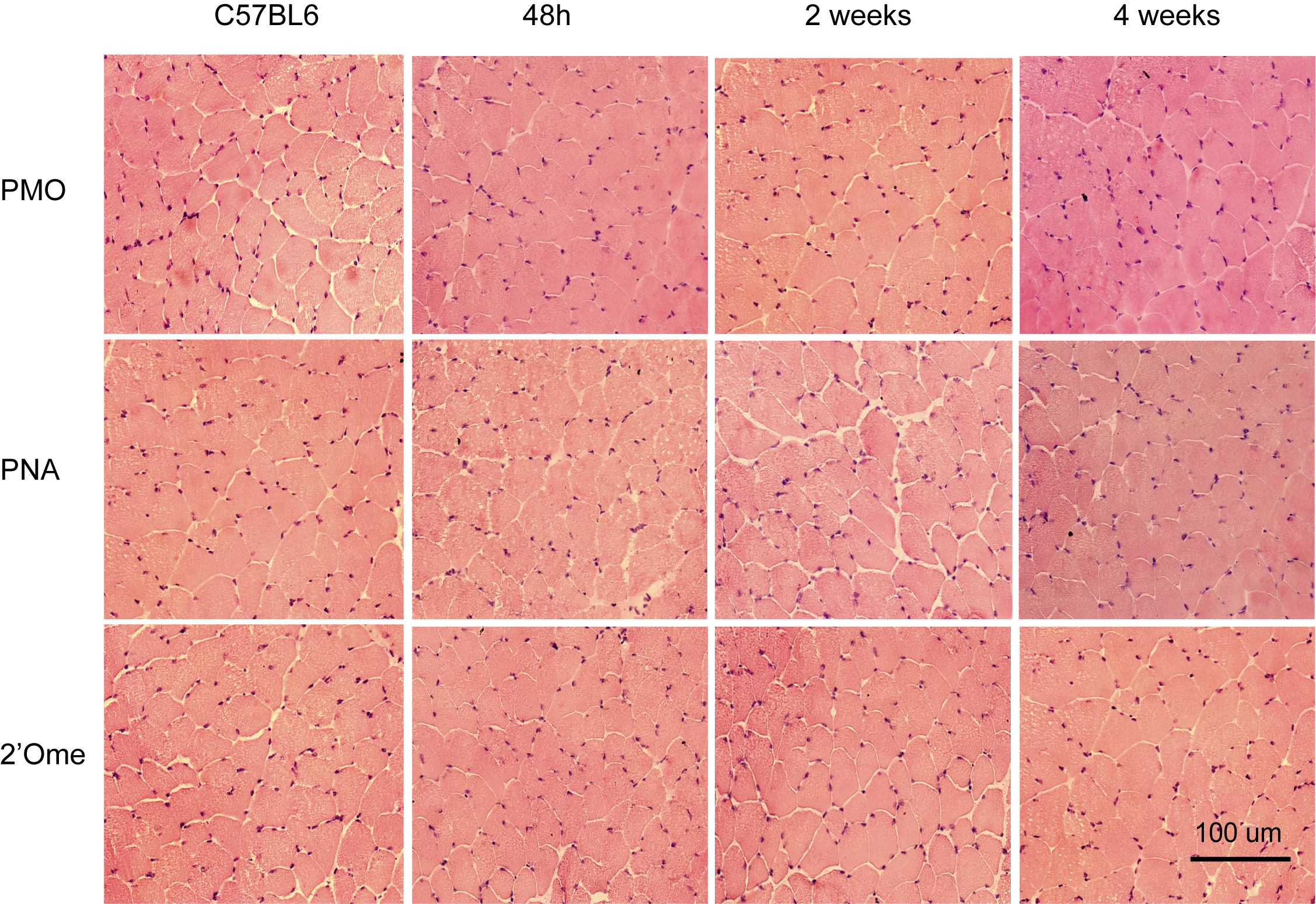

Supplement: Figure S2 — Routine hematoxylin and eosin staining for examining muscle morphology. Hematoxylin and eosin staining of TA tissue sections from treated C57BL6 mice with 2 µg PMO, 5 µg PNA and 5 µg 2′Ome PS by local injection at different time-points e.g. 48 hr, 2 and 4 weeks after injection, and C57BL6 normal controls. Scale Bar = 100 µm. No difference was observed between treated and untreated mdx mice. (TIF) [file pone.0111079.s002.tif]

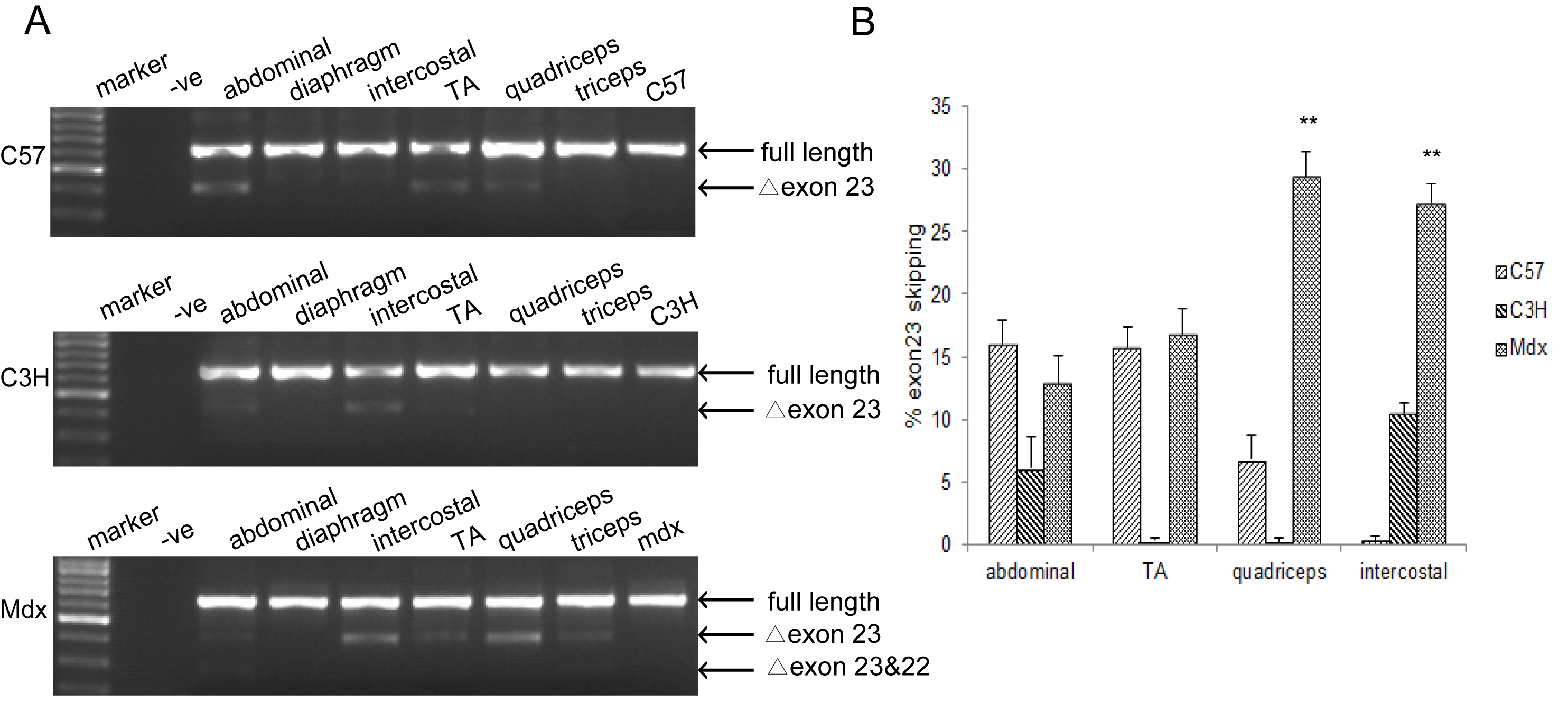

Supplement: Figure S3 — Systemic evaluation of PMO in wild-type and mdx mice. (A) RT-PCR results for systemic validation in C57BL6, C3H and mdx mice with PMO intravenously, at a low dose of 25mg/kg for 3 weekly injections. The numbered Δexon23 is for exon 23 skipping. (B) Quantitative evaluation of exon skipping in body-wide muscles in treated C57BL6, C3H and mdx mice (**P<0.001, n = 3). (TIF) [file pone.0111079.s003.tif]
