# Supplementary figures and images for: Correction: Wild-Type Mouse Models to Screen Antisense Oligonucleotides for Exon-Skipping Efficacy in Duchenne Muscular Dystrophy
Source: PLoS One. 2018 Nov 15;13(11):e0207817. doi: 10.1371/journal.pone.0207817 (PMC6237414; doi:10.1371/journal.pone.0207817)

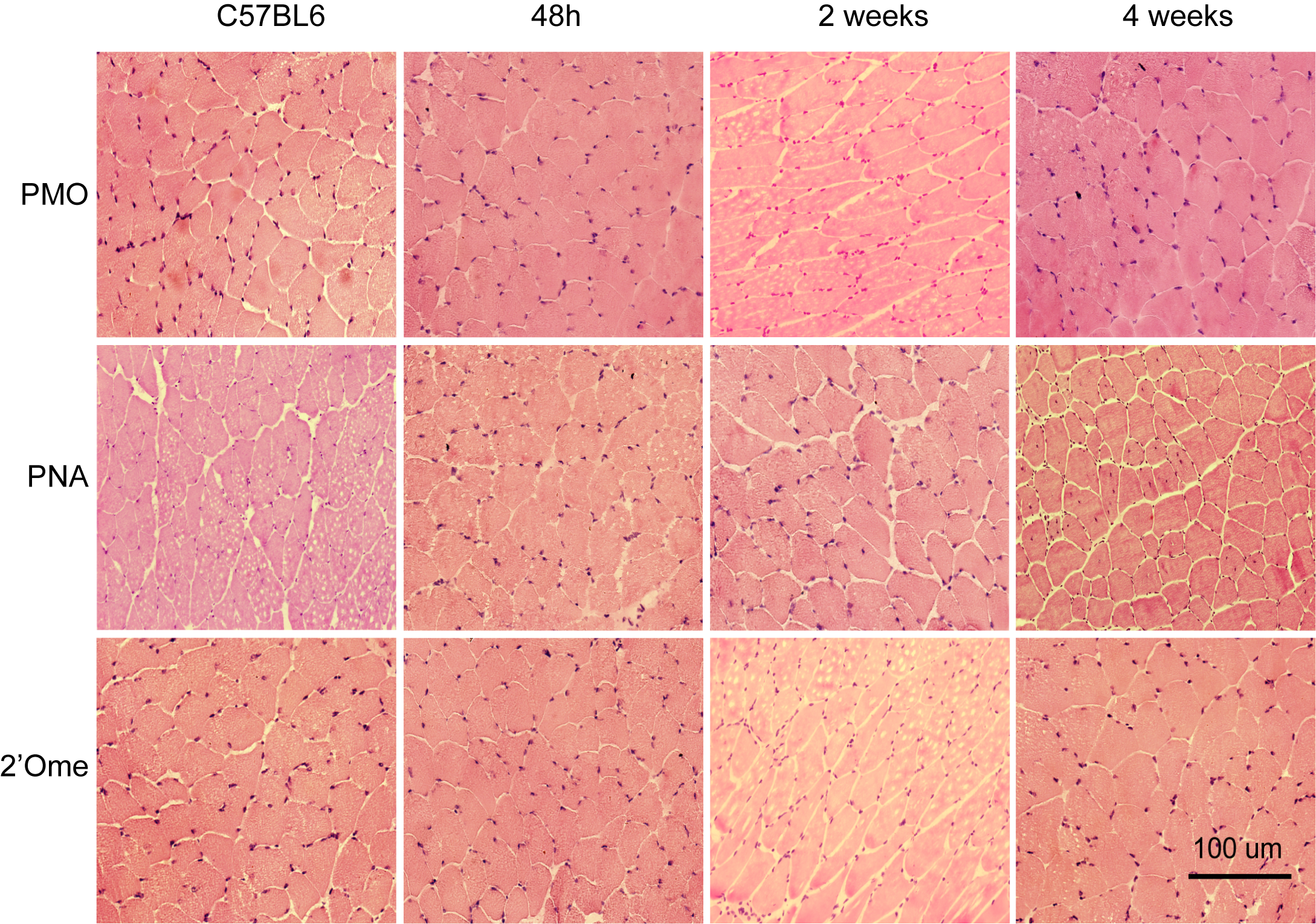

Supplement: S2 Fig — Hematoxylin and eosin staining of TA tissue sections from treated C57BL6 mice with 2 μg PMO, 5 μg PNA and 5 μg 2′Ome PS by local injection at different time-points e.g. 48 hr, 2 and 4 weeks after injection, and C57BL6 normal controls. Scale Bar = 100 μm. No difference was observed between treated and untreated mdx mice. (TIF) [file pone.0207817.s001.tif]
